# Editorial: Pathogenic *Neisseria*: Pathogenicity, vaccines, and antibiotic resistance

**DOI:** 10.3389/fcimb.2022.1119244

**Published:** 2023-01-05

**Authors:** Jesús Arenas

**Affiliations:** Unit of Microbiology and Immunology, Faculty of Veterinary, University of Zaragoza, Zaragoza, Spain

**Keywords:** *Neisseria meningitidis*, *Neisseria gonorrhoeae*, vaccines, antibiotic resistance, bacteria competition, mafA/B system, two partner secretion system

The genus *Neisseria* includes commensal species that form part of the flora of human and animal mucosa, but also includes two major pathogenic species: *Neisseria gonorrhoeae* and *Neisseria meningitidis. N. meningitidis* inhabits the upper respiratory tract and can cause meningococcal disease, a disease of rapid onset that involves sepsis and meningitis. *N. gonorrhoeae* lives in the genital, rectal and oral mucosa, and can cause gonorrheal infection that involves pelvic inflammatory disease, infertility, ectopic pregnancy, and neonatal blindness when transferred from an infected mother to the neonate during delivery. Recent discoveries expand our knowledge about interbacterial interactions, vaccine development and diagnostics.

Both *Neisseria* species are exclusively adapted to humans, therefore they evolved mechanisms to persist human defenses, acquire nutrients from the host and compete with the bacterial microbiome. Indeed*, in vivo* studies gained evidences that different *Neisseria* species can colonize a host at multiple sites of the nasopharynx and oral cavity (Sáez Nieto et al., 1998; Donati et al., 2016), and that *N. lactamica* prevented *N. meningitidis* colonization (Evans et al., 2011; Deasy et al., 2015), suggesting inter species competition. In the report by Baerentsen et al. competition mechanisms amongst *Neisseria* sp are summarized, comprising polymorphic toxins, bacteriocins and methylated DNA. Polymorphic toxins were discovered last decade and involve the Two Partner Secretion System (Arenas et al., 2013) and the MafA/B system (Arenas et al., 2015; Jamet et al., 2015). Both systems follow a similar genetic organization. However, TpsA and MafB toxins are structurally different, including secretion systems, toxin delivery, toxin processing, and protein production. Therefore its biological functions can substantially differ. But *Neisseria* can potentially produce bacteriocins or toxic metabolites, for example gonocins (Flynn and McEntergart, 1972) or meningocins (Kingsbury, 1966), which can also inhibit the growth of gonococcus or several *Neisseria*, respectively. The origin of these substances remains unclear but could help to discover antibiotic alternatives. A new and fascinating system is DNA methylation, which has been demonstrated to take place between *N. elongata* and *N. gonorrhoeae* or *N. meningitidis* (Kim et al., 2019). In the proposed model, DNA is transferred between bacteria, and the high degree of sequence homology allows multiple recombination. At these sites, methylation mismatch leading to restriction enzyme cleavage and chromosome degradation (Kim et al., 2019; So and Rendon, 2019).

Capsular and subcapsular commercial vaccines against *N. meningitidis* have been developed so far, and they cover the most relevant disease related serogroups (Pizza et al., 2020). However, commercial vaccines against *N. gonorrhoeae* are lacking, while the number of antimicrobial resistant clinical isolates is drastically increasing worldwide. High frequency phase and antigenic variation of surface exposed antigens appears to be one of the main drawbacks to promote vaccine development. This is illustrated in the work conducted by Shaskolskiy et al., who reported a comparative whole-genome analysis for *N. gonorrhoeae* isolates of genogroup 807, the most common in the Russian Federation, to other predominant genogroups worldwide. Authors found about 8-20 specific genes to each sequence type, including loci for phase variation and components of the gonococcal genetic island. Also, gene substitutions, mutations and absence in T4SS DNA secretion system encoding genes were detected. Remarkably, a variety of alleles of genes coding for pili proteins, transmembrane transporters, or components of MafA/B systems, amongst many others, were identified. Overall, clinical *N. gonorrhoeae* isolates expose a variety of structures at the surface, which makes difficult to find antigens to unsure cross protection in a universal vaccine. Maurakis and Cornelissen revised the most recent studied antigens, including TonB dependent transporters, lipo-oligosaccharides epitopes, and OMVs based or bacterial ghost vaccines. The transferrin binding protein A and B were largely studied, also in *N. meningitidis*, because of conservation, surface exposition and immunogenicity. But membrane proteins undergo problems for antigen production and stabilization. To overcome these issues, successful hybrids fusing TbpA loop 2 to the N-terminal lobe of TbpB were generated and elicited protective antibodies (Price et al., 2007). Besides, some LOS epitopes, such as L8 or 2C7, which are conserved amongst gonococci, resulted immunogenic, and stimulated bactericidal IgG responses in mice (Gulati et al., 1996; Ram et al., 2018; Gulati et al., 2019). Antigen platforms were also examined, including OMVs based vaccines, which had shown good results in the generation of meningococcal vaccines. Examples include IL-12 encapsulated OMVs (Liu et al., 2018), fHBP overexpression OMVs and an attenuated lipid A OMVs (Beernink et al., 2019) or detoxified meningococcal OMVs (Kathryn et al., 2022). Also, bacterial ghost, which are empty shells, are being used for antigen delivery such as NspA (Jiao et al., 2021).

Rapid and accurate diagnosis is critical for timely treatment of Neisserial infections. In line with this, spherical goal nanoparticles with shorth single DNA strand linked at the particle surface were developed by Carter et al to rapidly identify gonococcal DNA. The probe DNA is complementary to gonococcal DNA uptake sequences, highly abundant in gonococcal genomes. They can hibridate gonococcal DNA and induces particle aggregation that can be colorimetrically detected. Identification of gonococcal DNA in samples from patients could takes about 30 min, and thus can be a fast and routinary technique. But, also, rapid detection of antibiotic resistance is critical for adequate treatment of Neisserial infections. *N. gonorrhoeae* rapidly develop antimicrobial resistance, including to sulfonamides, penicillins and fluoroquinoles (Lorenzo-Lurenco et al., 2017; Aitolo et al., 2021). Ciprofloxacin is a quinolone used for treatment of meningococcal and gonococcal infection. Ciprofloxacin activity is based on the its interaction with DNA gyrase and topoisomerases. Ciprofloxacin resistance is attributed to mutations in target genes, i.e. *gyrA* or *parC* genes that codes for subunits of gyrase and topoisomerase. The gold standard technique to detect the origin of ciprofloxacin resistance is sequencing, but it is time consuming. To overcome this issue, a rapid test combining mismatch PCR targeting *gyrA* and subsequent digestion patterns of PCR products with *Aci*I was proposed by Ota et al. This method based on the detection of T91I mutation in *gyrA*, one of the most common mutations that confer ciprofloxacin resistance. Identification of ciprofloxacin can take 4 hours and does not require bacterial growth.

In summary, the work published here reinforces the knowledge about two pathogenic species and the development of novel techniques for diagnosis and prevention. I hope that this Research Topic will stimulate new research in this field where comprehensive molecular mechanisms remain to be elucidated.

## Author contributions

JA (University of Zaragoza, Spain) edited this Research topic and wrote the manuscript. The author contributed to the article and approved the submitted version.
